# Artificial intelligence orchestration for text-based ultrasonic simulation via self-review by multi-large language model agents

**DOI:** 10.1038/s41598-025-97498-y

**Published:** 2025-04-11

**Authors:** Soyeon Kim, Yonggyun Yu, Hogeon Seo

**Affiliations:** 1 Korea Atomic Energy Research Institute, 111, Daedeok-daero 989beon-gil, Daejeon, 34057 Republic of Korea; 2 University of Science & Technology, 217, Gajeong-ro, Yuseong-gu, Daejeon, 34113 Republic of Korea

**Keywords:** Simulation, Large language models, Multi-agents, Natural language commands, Text-based control, Automation, Mechanical engineering, Computer science, Information technology

## Abstract

Widely used ultrasonic simulation systems often rely on complex graphical user interfaces (GUIs) or scripting, resulting in substantial time investments and reduced accessibility for new users. In this study, we propose a novel text-based simulation control architecture, which leverages a large language model (LLM) and the ground artificial intelligence (AI) approach to streamline the control of ultrasonic simulation systems. By modularizing the functionalities of the SimNDT program into discrete functions and enabling natural language-based command interpretation, the proposed method reduces the average simulation configuration time by approximately 75%. To further mitigate task failures in scenario generation using the LLM, we introduce the ground AI approach, which employs self-review mechanisms and multi-agent collaboration to improve task completion rates. In particular, when vectorized output lengths deviate from the standard, we regenerate outputs using multiple LLM agents, reducing the scenario generation error rate from 23.89 to 1.48% and enhancing reliability significantly. These advancements underscore the potential of AI-driven methods in reducing operational costs and enhancing reliability in simulation frameworks. By integrating text-based control and Ground AI mechanisms, the proposed approach provides an efficient and scalable alternative to traditional GUI-based control methods, particularly in time-sensitive applications such as digital twin systems.

**Supplementary Information:**

The online version contains supplementary material available at 10.1038/s41598-025-97498-y.

## Introduction

In engineering, simulations are indispensable tools for analyzing and predicting the behavior of complex systems. They are widely utilized in fluid dynamics, structural analysis, and energy systems to support decision-making and enhance design processes. However, generating large amounts of diverse simulation data for practical applications requires expert manipulation and remains challenging owing to the inherently time-consuming and complex nature of repetitive simulation control. Prevailing methodologies rely heavily on graphical user interfaces (GUIs), necessitating manual user interaction, which limits automation and scalability when extensive datasets are required^1–6^.

Integrating large language models (LLMs) into simulation workflows offers the potential to address these challenges. LLMs, such as the generative pre-trained transformers (GPTs)^7,8^, have demonstrated remarkable capabilities based on training on extensive datasets, enabling rapid and efficient text processing^9^. By leveraging LLMs for natural language-based simulation control, repetitive tasks may be automated, parameter tuning may be optimized, and data generation processes may be streamlined significantly. This approach could revolutionize simulation management, enhancing efficiency and scalability across various engineering applications.

To this end, we propose a text-based simulation control framework by leveraging LLMs. By modularizing the functionalities of SimNDT: Ultrasound NDT simulation tool (Version: 0.52)^10^ into callable functions, we enable users to control simulations via natural language commands. For instance, when a user might input: ”Simulate ultrasonic wave propagation in a rectangular object with a width of 10 and a height of 5,” the system interprets the prompt and executes the corresponding simulation scenario. This novel method simplifies simulation control and facilitates more intuitive interactions compared to traditional GUI-based methods. However, despite initial results suggesting significant improvements in efficiency and usability, challenges such as those regarding accuracy and reliability of LLM-generated scenarios persist. To address these, we develop strategies based on Ground artificial intelligence (AI), which enhances LLM-driven systems by incorporating self-review mechanisms and multi-agent strategies to improve decision-making accuracy and reduce errors^11^. In this approach, LLMs refine their outputs iteratively or collaborate with other agents to generate more reliable results, providing a robust framework for addressing their inherent limitations in complex tasks.

In this work, we propose a novel text-based simulation control method that draws on large language models to address the constraints of traditional GUI-based approaches. We also develop a systematic integration of Ground AI principles-ranging from single-agent methods to multi-agent collaborations-and demonstrate how these strategies can reduce inaccuracies in LLM outputs. Furthermore, we validate the practical feasibility of our framework by incorporating it into SimNDT and assessing its performance under realistic ultrasonic wave propagation scenarios. By integrating sophisticated AI methodologies with ultrasonic simulation, this research presents a scalable, efficient alternative for managing complex engineering simulations, thereby expanding the potential of AI-powered tools in computational science and engineering.

The remainder of this paper is organized as follows. The implementation of the proposed LLM-based text simulation control architecture is discussed in Section 2, besides the text command-based approach, the modular structure for LLM function calls, and practical examples of text-based control. In Section 3, error reduction techniques based on the Ground AI approach are described, along with the concept, experimental configuration, and results of various error mitigation strategies. The overall results are presented and discussed in Section 4, highlighting the contributions and implications of our work. Finally, the paper is concluded in Section 5 by summarizing key findings and suggesting future research directions.

## Literature review

Interest in AI models and their utilization^12,13^ have steadily increased over the past few years, spanning a wide range of industries, including economics and healthcare. The emergence of LLMs, e.g., the attention^14^-based architectures, BERT^9^ and GPT^7,8^, has not only motivated significant advancements in AI performance but also accelerated efforts to integrate these models within industrial applications. Organizations and research institutions are actively adopting large language models to achieve objectives such as increased productivity, cost reduction, personalized customer services, and precision medicine. The scope of real-world applications of LLMs is expanding rapidly, from medical diagnosis support systems and financial analysis to automated translation and AI-driven customer service chatbots^12,15,16^. This trend transcends mere AI adoption; it is fundamentally reshaping industrial processes and serving as a catalyst for innovation across sectors^17–19^. However, as AI technology continues to evolve, new concerns regarding ethics, privacy, and algorithmic bias are emerging. Addressing these concerns requires the establishment of proper guidelines to ensure transparency and implement responsible AI operations.

Consequently, AI, and in LLMs with their enhanced expressiveness and learning capabilities in particular, has evolved from merely serving static functions into a crucial tool for creative problem-solving and complex decision-making. This trend is expected to accelerate further in the coming years, leading to the continuous expansion of the scope of AI applications and profound transformations in various industries^11,15,16,20^. With the advancement of AI, autonomous controlling systems on this basis have gained prominence, leading to the development of the concept known as ’orchestration.’ Orchestration refers to the process of coordinating and managing the deployment, integration, and interaction of various AI components. Additionally, LLMs are assigned roles specific to individual agents, facilitating collaboration between humans and AI (often referred to as human-AI interaction) and expanding research related to agents. This approach enables AI agents to act autonomously and perform complex tasks without human intervention. Thus, AI orchestration and agent-based methodologies enhance the autonomy and efficiency of AI systems, elevating human-AI collaboration^21–31^ to a new dimension.

Traditional simulation automation frameworks^11,12,15,20,32^, such as MyCrunchGPT^19^ and the AI-based design system^32^, have made significant progress in data analysis, model recommendations, and single-step optimization. However, they lack a structured framework capable of fully orchestrating iterative simulations in a feedback-driven manner^26^. MyCrunchGPT leverages LLMs for scientific machine learning tasks, primarily focusing on data analysis and model selection rather than on direct simulation execution. Similarly, the approach proposed by Park et al. employs a single-model generative AI pipeline integrated with an optimization solver, but does not incorporate a multi-agent system^16,33,34^, limiting its ability to conduct continuous, feedback-driven iterative experimentation.

In contrast, the framework proposed in this paper adopts a multi-agent architecture, where one agent translates natural language input of the user into executable commands, another executes the simulation based on these commands, and a third evaluates the results to refine inputs for subsequent iterations^28^. This automated workflow transcends single-step analysis or static optimization by continuously leveraging previous simulation results to guide subsequent iterations. Unlike existing approaches that operate within a fixed input-output loop, the proposed framework ensures that iterative feedback influences future simulation conditions dynamically, enabling a more autonomous and self-improving process. Moreover, the proposed framework adopts a modular orchestration strategy, allowing LLMs to control each stage of the simulation pipeline rather than being restricted to auxiliary functions. This design enhances simulation flexibility by enabling the framework to incorporate responses from another model automatically to refine the decision-making process when an intermediate result is determined to be erroneous^23^. This self-review mechanism not only improves simulation reliability but also significantly reduces the need for human intervention for error handling and adjustments. As a result, the proposed framework fosters a more efficient, robust, and autonomous simulation automation environment. Table 1 provides a comparison between existing research approaches, their limitations, the necessity of our work, and our proposed methods that overcome these challenges through structured function calling, schema-based validation, and self-review mechanisms.

**Table 1:** Flow of developments: existing research limitations and our improvements.

Existing research	Limitations	Necessity of our work	Proposed method (ours)
Transformer-based LLM^7–9,14^	High computational cost and complexity; issues with factual accuracy, reasoning, and biases	Need for accurate translation from natural language to structured commands to mitigate misinterpretations	Using GPT-4o with structured function calling and predefined simulation schemas to improve accuracy and reduce ambiguity
Human-AI interaction (HAI) basedprogram control^21,22,24,27^	Limited real-world applicability and insufficient structured command handling; limited generalization and robustness	Robust, structured, and verifiable command interpretation required for practical program control	Employing structured prompt inputs and LLM-driven function calling mechanisms combined with schema-based validation
Multi-Agent and autonomous AI systems^16,25,26,28^	High complexity, limited resource efficiency, poor generalization across diverse scenarios	Efficient single-agent solutions needed for reliable scenario generation and resource optimization	Integrating Ground AI verification with single and multi-LLM agent configurations to ensure reliability while controlling resource usage
Human-feedback based learning^29–31^	Limited scalability and generalization to complex tasks; lack of structured command verification	Automatic validation and self-correction of structured commands required for complex tasks	Self-review mechanisms and structural schema validation embedded in LLM-generated function calls
AI-based simulations^12,13,19,32^	Complexity in simulation setup, limited accessibility, insufficient verification of simulation parameters	Simplified and accessible methods for configuring and running accurate simulations needed	Prompt-based natural language inputs mapped automatically via Simulation Variable Schema to structured executable commands
Language-based data visualization andAI tools^15,17,18,23^	Limited handling of complex computational tasks; insufficient real-world validation	Effective integration of natural language commands with computational tasks, providing realistic and validated results	Structured parameter generation via predefined schemas linked directly to computational tasks within simulations

## Implementation of LLM-based text simulation control architecture

To control the program using LLMs, we modularize the various functionalities of the SimNDT program into callable functions and design a code-based controllable structure. This enables the programmatic control of multiple tasks, including simulation configuration, execution, and result output. In this work, we adopt a modular architecture for the SimNDT program by separating core functionalities into distinct classes or functions. Specifically, components such as Scenario, Material, Boundary, Transducer, Signal, and Simulation are each implemented as individual modules, which are collectively packaged based on a SimPack object. Using this design, an LLM (e.g., GPT-4o) provides natural language inputs that are subsequently mapped to the corresponding modules, thereby orchestrating simulation tasks, such as configuration, execution, and output handling in a step-by-step, programmatic manner. Further, low-level details, such as the physics engine or postprocessing routines, are encapsulated under the SimNDT.engine folder, allowing independent functionalities, e.g., the EFIT2D solver, to operate without directly coupling with the high-level control. In practice, higher-level classes, such as EngineController or SIM Custom, coordinate with these modules to run and manage the simulation. As a result, the LLM-based interface focuses on parameter delivery and orchestration logic, while the actual computation or physics modeling is handled by separate, specialized modules. This code-based structure not only enhances flexibility and scalability in ultrasonic simulation but also simplifies the extension to other domains (e.g., thermal or structural analysis) if needed.

With this modular design, we implemented an environment that interprets natural language text commands using the GPT-4o model of OpenAI to orchestrate simulations. Users can execute simulations based on simple text inputs without complex code or GUI operations, greatly enhancing user experience. LLMs translate the input natural language commands into SimNDT function calls, and the simulation engine executes them to yield the results.

### Text command-based simulation control architecture

**Figure 1:**
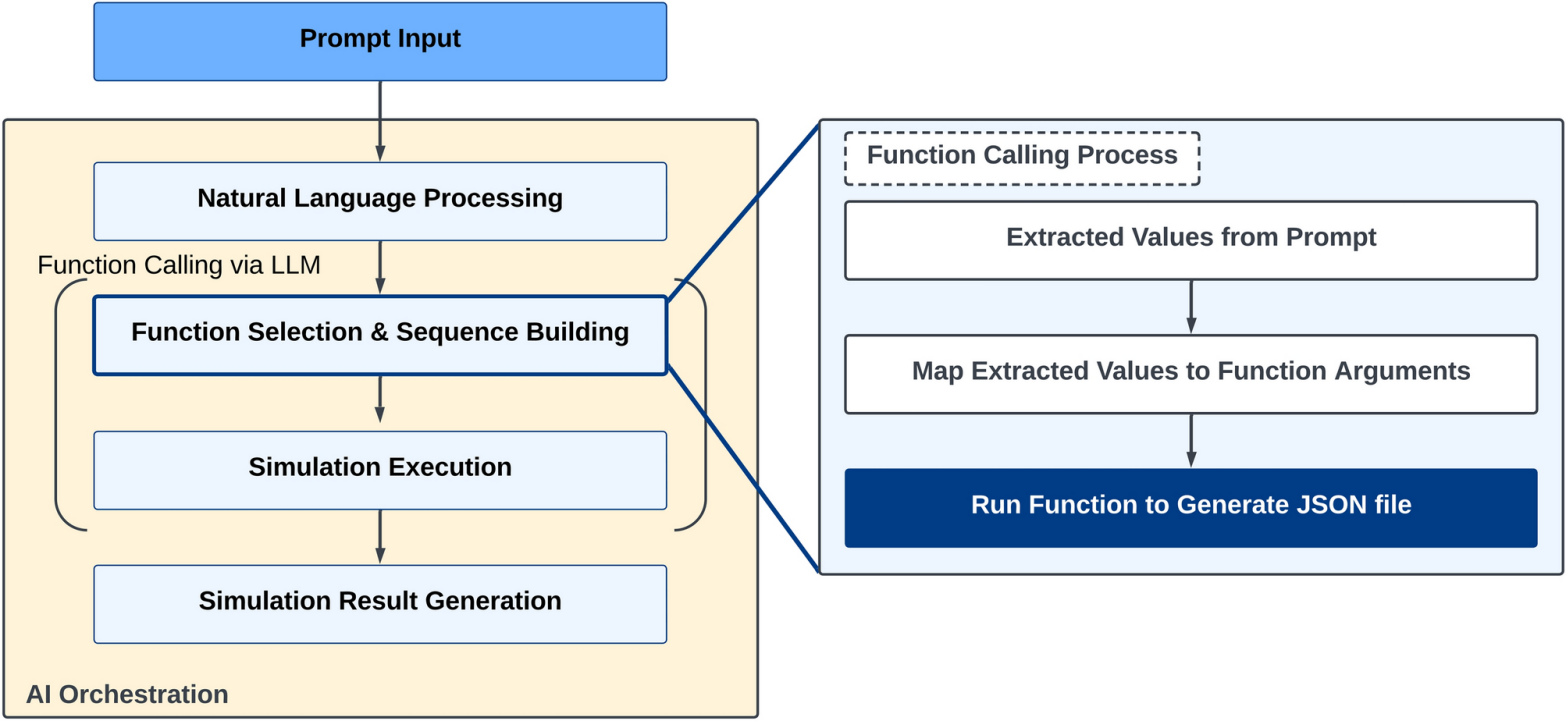
Flowchart of text command-based simulation control using LLM.

The text command-based simulation control comprises three primary components—prompt input, LLM, and the simulation engine. The entire process is depicted in Fig. 1. The prompt input is the medium in which users enter text prompts, i.e., natural language commands that specify the desired simulation tasks. Using the prompt input, users can easily instruct the system on the simulations they wish to perform without the need for complex coding or GUI navigation. LLM functions as the interpreter within the text-based simulation control framework. It processes the input text commands and translates them into code or specific function calls comprehensible to the simulation engine. This translation is crucial as it bridges the gap between human language and machine-executable instructions, enabling seamless interaction between the user and the simulation engine. Finally, the simulation engine receives the code or function calls generated by LLM and executes the corresponding simulations, returning the results to the user. This component performs the computational tasks required for the simulations, serving as the core component of the system that delivers the outcomes to the user. Integrating these components allows users to control simulations using simple text prompts, simplifying the simulation configuration and execution processes significantly.

**Algorithm 1:**
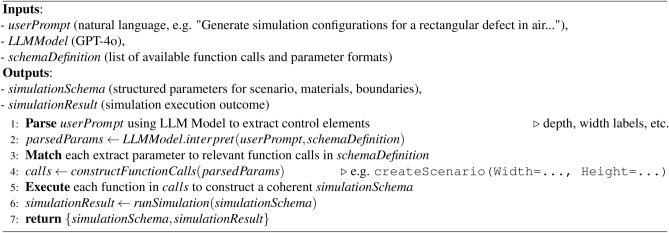
LLM-driven simulation configuration and execution

### Program control through LLM function calling structure

To control the program effectively, we employ the function calling mechanism provided by the LLM, GPT-4o. This feature enables the LLM to generate structured data that adheres to predefined function signatures, facilitating seamless integration between natural language inputs and programmatic function calls. In the implementation described here, the SimNDT program is transformed into a set of modular functions, each corresponding to a specific simulation task. To this end, a Simulation Variable Schema is developed as shown in Supplementary Figures (Figs. S1-3), which describes the variables necessary for these functions and facilitates their functionization. The schema includes comprehensive descriptions for each variable, which help the LLM understand the intended purpose and utilization of each variable, enhancing the relevance of the generated function calls.

**Figure 2:**
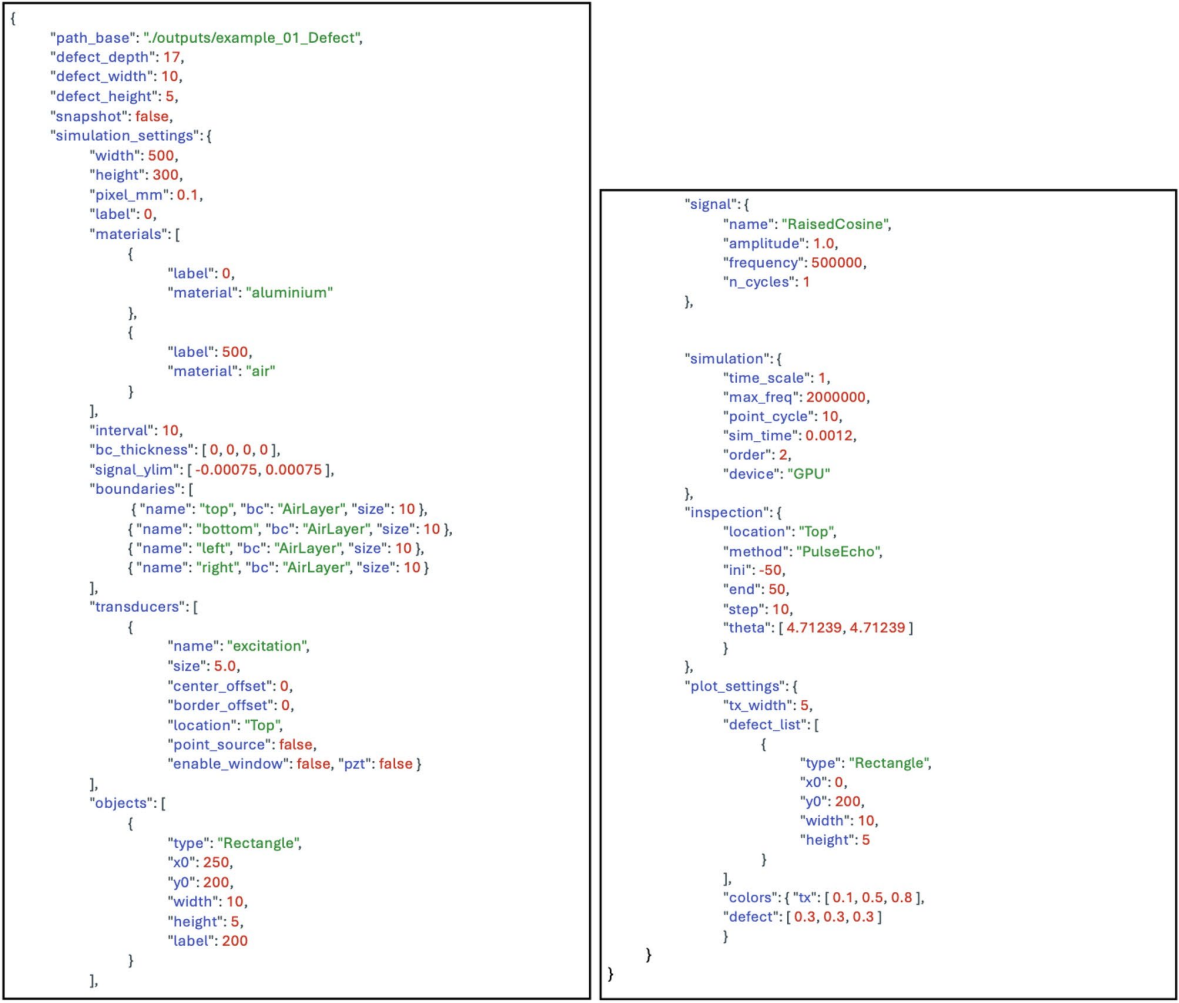
Example of an output simulation scenario generated using function calls.

Upon receiving a natural language command from the user via a prompt, the LLM initiates input processing. The function calling mechanism generates a function call corresponding to one of the predefined functions. Subsequently, the model identifies the most suitable function and populates the parameters following the input of the user, reflecting the intended outcome. If the user does not specify particular parameters, the model utilizes default values defined within the simulation variable schema to guarantee that the function call is complete and executable. For instance, if a user inputs the command ”Simulate ultrasonic wave propagation in a rectangular object with a width of 10 and a height of 5,” the LLM interprets this command and generates the corresponding simulation scenario via function calling based on the simulation variable schema, as illustrated in Fig. 2. This structured output follows the predefined simulation variable schema and can be directly utilized by the simulation engine to execute the simulation.

The proposed architecture enables the parallel generation of multiple simulation scenarios during the LLM processing stage. However, in the simulation execution phase, support for dedicated computational acceleration (such as GPU parallelization or cluster environments) is currently limited. Therefore, the feasibility of parallel execution depends on the complexity of the simulations and the available resources. Thus, while the architecture allows for the efficient simultaneous creation of various simulation scenarios, the actual execution phase may require sequential processing depending on the circumstances.

The simulation variable schema enumerates all essential parameters, e.g., geometry definitions, material properties, boundary conditions, and solver configurations, ensuring that each function call includes every component required for a valid simulation. If certain parameters are omitted in command of the user, they are automatically populated with predefined defaults specified by the schema, preventing incomplete or invalid configuration requests. Moreover, users can easily override default values using subsequent prompts or edits if they wish to refine specific conditions (e.g., adjusting the wave frequency or boundary thickness). This approach not only streamlines the configuration process for novices but also provides experienced users with precise control, striking a balance between simplicity and flexibility. As a result, once the schema-based function call is finalized, it can be transmitted to the underlying simulation engine seamlessly for immediate execution.

By leveraging the function calling mechanism, the LLM generates outputs that conform to the expected format consistently, thereby reducing errors and enhancing the stability of simulation execution. Further, this approach enables the LLM to comprehend the variables intended to be set by the user based on their input, thereby improving the validity and reliability of the simulation scenarios generated by the LLM. Furthermore, the simulation variable schema is constructed using the principles of functional modularization. The description field within the schema included an explanation of the function and role of each variable. This helps the LLM understand the meaning of each variable, enabling the selection of suitable parameter configurations based on the input of the user. Default values are assigned in anticipation of scenarios where users may not set specific variables. This guarantees that all essential parameters for simulation execution are perpetually accessible, thereby enhancing the robustness of the text-based simulation control framework. Due to this structured approach, the LLM generates outputs following the predefined simulation variable schema consistently with each call, thereby ensuring stability in simulation execution. The LLM accurately interpreted the input of the user to determine which variables they wished to set and generated simulation scenarios accordingly for program execution.

**Figure 3:**
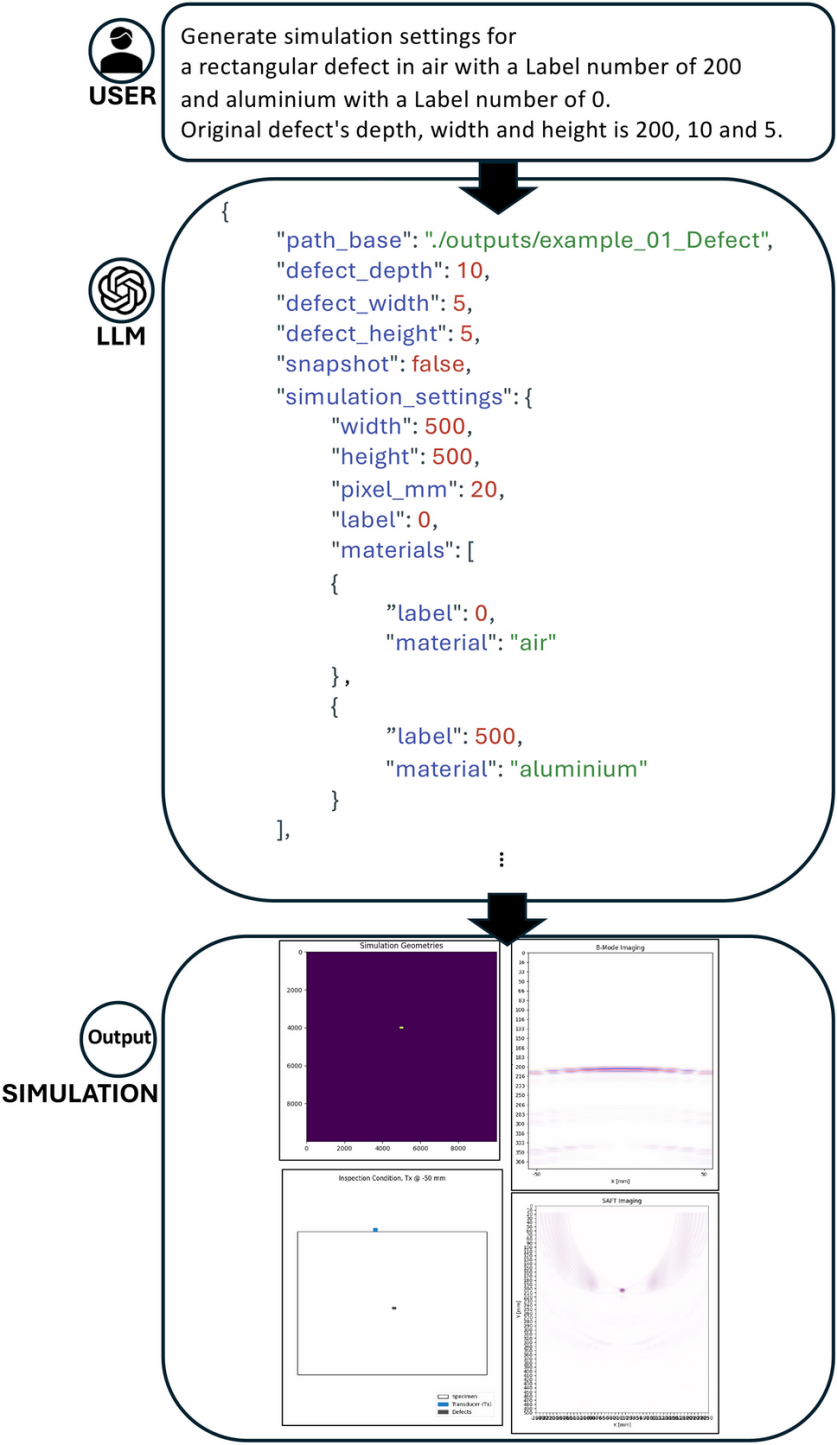
Process flow of text-based command execution and simulation outputs.

### Efficient text-based control and examples

The proposed system is an effective control tool, facilitating user interaction based on natural language prompts. For instance, a user may input: ”Generate simulation configurations for a rectangular defect in air with a label number of 200 and an aluminum background with a label number of 0. The defect’s depth, width, and height are 200, 10, and 5, respectively.” The LLM interprets the natural language commands and generates the requisite code or function calls for execution. Subsequently, the simulation engine executes the simulations and furnishes the results to the user. The outputs include the simulation geometry image, the simulation inspection condition animation, and the ultrasonic simulation result data stored as npy files. The npy files represent the ultrasonic simulation results stored as 2D matrices. Each value in the matrix corresponds to the signal amplitude at a specific spatial coordinate. Based on the aforementioned data, supplementary images, such as B-mode and synthetic aperture focusing technique (SAFT), are generated in the PNG format. The B-mode and SAFT graphs are generated based on the data obtained from the npy files, which visually represent the simulation results, as depicted in Fig. 3.

Implementing the proposed text-based control system reduces the time required for the configuration and execution of ultrasonic wave propagation simulations using the SimNDT program significantly. The average time required to execute a particular simulation is observed to decrease from approximately two minutes, required by the traditional GUI-based control method, to approximately 30 seconds, required to write a prompt for the control command. This represents a 75% reduction, thereby greatly enhancing overall efficiency. In turn, this streamlines the workflow, rendering the system particularly useful when large amounts of simulation data are required or when continuous execution of simulations is necessary. Thus, the execution of numerous simulations over a short period becomes more feasible, which is especially beneficial in applications that demand repeated simulations.

## Error reduction based on ground AI and experimental results

### Concept and implementation of ground AI

Grounded AI represents an approach to ensuring that outputs generated by AI models are firmly based on reliable evidence rather than hallucinations or unfounded assertions. Despite their extensive training on diverse datasets, large language models may produce responses that appear plausible but lack factual accuracy. Ground AI addresses this limitation by implementing verification mechanisms that either validate outputs against trustworthy data sources or incorporate self-correction protocols, thereby minimizing potential issues such as misinformation or logical inconsistencies in AI-generated content^35,36^ A detailed comparison between rule-based validation and Ground AI approaches is summarized in Table 2, highlighting their differences in algorithmic flexibility, error management strategies, and contextual intelligence.

**Table 2:** Comparative analysis of validation methodologies for rule-based and ground AI approaches.

Criteria	Rule-based validation	Ground AI approach
Validation paradigm	Static and predefined rule enforcement	Dynamic and adaptive multi-agent verification
Algorithmic flexibility	Constrained by predetermined logical conditions	Adaptive inference with contextual recalibration
Error management strategy	Deterministic binary compliance checking	Iterative error detection and generative correction
Computational complexity	Low algorithmic overhead	Moderate computational complexity with scalable agent configuration
Scenario adaptation capability	Limited domain-specific applicability	High contextual generalizability
Output refinement mechanism	Strict output rejection	Intelligent regeneration and systematic improvement
Verification protocol	Deterministic checklist-based matching	Structural integrity validation with probabilistic enhancement
Contextual intelligence	Minimal semantic understanding	Advanced contextual reasoning and interpretative capabilities
Architectural scalability	Challenging cross-domain implementation	Modular design enabling efficient domain transposition
Artificial intelligence integration	Minimal AI model engagement	Comprehensive leveraging of large language model capabilities

**Figure 4:**
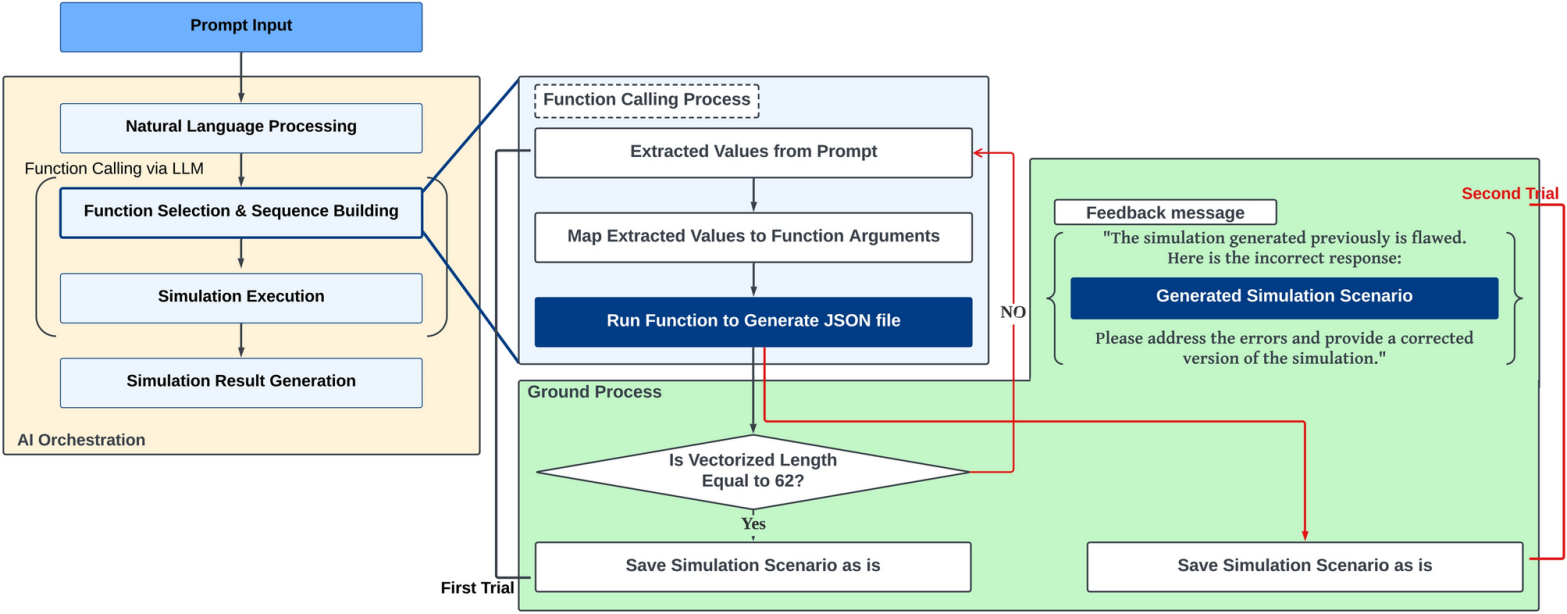
Flowchart of ground AI with self-review (single agent) approach.

In this paper, Ground AI is employed to mitigate failures in the scenario generation process during simulation control. Considering a single-LLM agent without Ground AI as the baseline, three distinct approaches are implemented by combining Ground AI with a single-LLM agent with self-review and multi-LLM agents. The self-feedback method based on the single-LLM agent entails prompting the LLM to review the answer and correct it if required, encouraging it to revise and regenerate its response, as illustrated in Fig. 4. In this study, the self-review process of the proposed Ground AI approach focuses on the structural validation of the generated simulation schema, without incorporating feedback based on simulation results. Specifically, the generated schema is transmitted along with an internal self-review prompt to verify and enforce compliance with requirements, such as the presence of essential fields and parameter consistency. In all Ground AI approaches, a verification process of LLM answers is applied to ensure the integrity of the simulation scenarios. Following the vectorization of the generated simulation scenario, the vector length is evaluated to ascertain its equivalence with the number of keys present in the simulation variable schema (62 schema keys in this study). If the vector length equals the number of keys, the scenario is deemed valid and utilized to execute its corresponding simulation. Conversely, the scenario is classified as invalid and discarded if this equivalence is not established.

Similarly, a structured verification mechanism is also employed in AI-powered structured query language generation platforms, e.g., Vanna.ai^37^, which validates the generated queries by ensuring that all required components are included. Taking inspiration from this approach, our study adopts a predefined schema of 62 keys to evaluate the completeness of AI-generated simulation scenarios. By verifying whether each scenario vector satisfies the 62-length criterion, we uphold a robust check against missing or inconsistent parameters, thereby enhancing both the reliability and reproducibility of the AI-driven simulation process. In the self-feedback method based on the single-LLM agent, if the verification is not passed, the system transmits the invalid scenario, the prompt of the user, and a review message indicating that the response was incorrect^38^. This message instructs the LLM to correct the response and regenerate the simulation scenario to address the errors and enhance the validity of the generated scenarios, as shown in Fig. 4.

**Figure 5:**
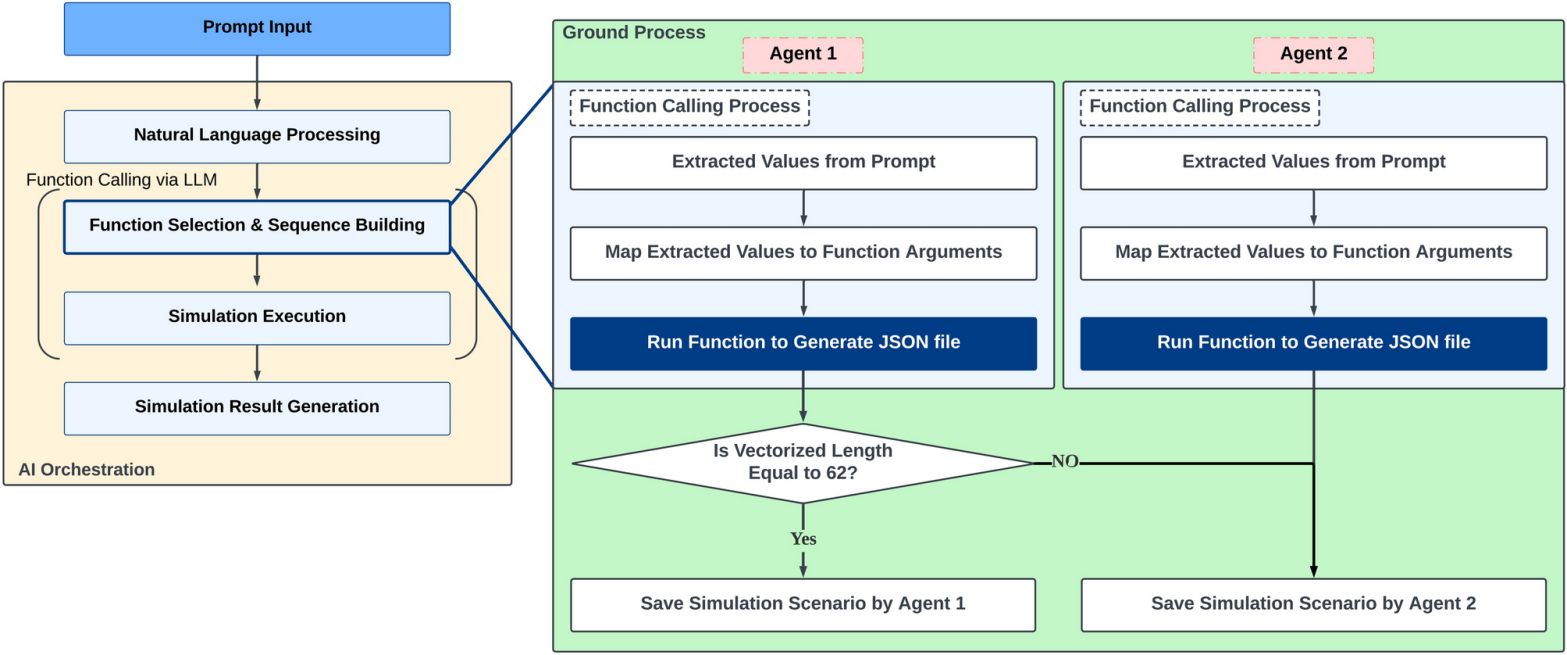
Flowchart of ground AI with multi-LLM agents (two agents) approach.

**Figure 6:**
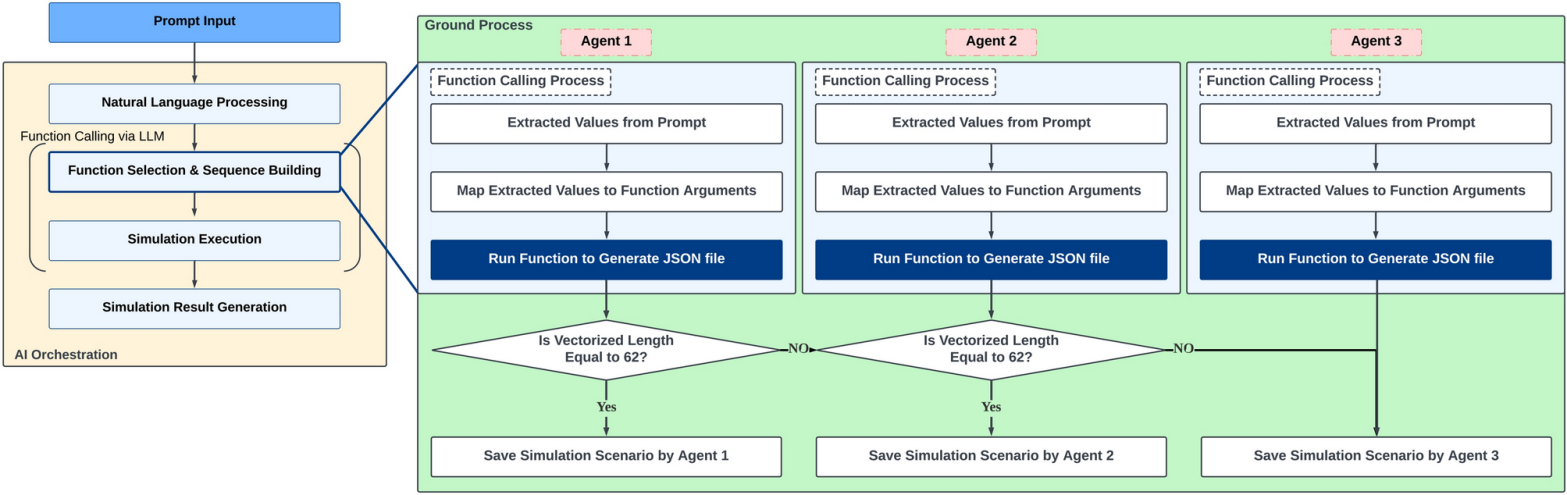
Flowchart of ground AI with multi-LLM agents (three agents) approach.

In contrast, multi-agent methods entail the utilization of multiple agents to generate responses in parallel. Each agent independently creates a simulation scenario, and these scenarios are validated in the order of generation. If the scenario from the initial LLM agent is deemed valid, its response proceeds to the subsequent phase, and the remaining agents remain inactive. If the initial scenario proposed by the first agent is deemed invalid, the scenario proposed by the second agent is validated next. This process is repeated until a valid scenario is generated, thereby ensuring the generation of a valid scenario. This method reduces the probability of failure by increasing the probability of obtaining at least one valid scenario based on the combined efforts of multiple LLM agents. However, multi-agent methods can become computationally expensive if excessively many agents are employed. Accordingly, the number of agents should be optimized to achieve an equilibrium between the computational cost and the probability of generating a valid scenario. Multi-agent methods with two and three agents are implemented to investigate the effectiveness of the number of LLM agents in Ground AI, as illustrated in Figs. 5 and 6, respectively.

### Experimental configuration

In our experimental design, we aimed to rigorously assess the capability of LLMs to generate complex simulation scenarios while adhering to strict structural requirements. The fundamental challenge lies not just in understanding user requests, but in consistently generating a specific, deep, hierarchical JSON schema. This schema, which serves as the input for simulation execution, mandates a precise structure with nested parameters across multiple levels (e.g., scenario configuration, material properties, boundary conditions, defect specifications). The task demands more than just plausible variable naming and value generation; it requires precisely replicating the predefined schema structure in its entirety for every generation, incorporating only the modifications specified by user input, while ensuring the generated values maintain physical consistency and adhere to simulation constraints. For instance, the defect specification alone necessitates coordinating multiple interdependent parameters (shape, dimensions, position, material properties) within a specific nested hierarchy, demonstrating the intricate structural integrity the LLM must preserve. Any deviation from this required structure results in a simulation input error.

Experiments are conducted to evaluate the performance of four approaches—the initially designed approach without Ground AI, and the methods incorporating the self-feedback mechanism of Ground AI using a single-LLM agent and multi-agent techniques using two and three agents. The performances of these methods are compared by adjusting a single variable in the simulation scenario to assess their impact on error rates in scenario generation.

Let us consider the following text control command (prompt) used for program control: ‘Generate simulation configurations for a rectangular defect in aluminum with a label number of 0 and air with a label number of 500. The original defect’s depth, width, and height are 1, 10, and 5, respectively.’ We aim to control the depth variable by assuming that it changes in increments of 1 mm. Even this simple modification of the user requirement, i.e., whether the LLM can create the simulation scenarios without failures, is investigated. On this basis, the failure probability of autonomous simulation control using the LLM and the periodicity can be evaluated qualitatively. To specifically probe the robustness of each approach against the difficulty of modifying this complex, fixed structure, we designed experiments with incremental modification demands based on user requirements. In the first experiment, only the depth of the defect is varied incrementally, whereas in the second experiment, both the depth and the width are varied. This dual-parameter modification significantly escalates the challenge, demanding that the LLM not only adjust multiple interdependent values but do so while meticulously preserving the integrity of the entire, complex, and predefined JSON structure. This stepwise approach allows for an assessment of the ability of the LLM to handle increasingly complex parameter changes and to understand the conditions under which simulation generation might fail or succeed.

All outputs are presented in the simulation scenario JSON results in the case of successful execution of the simulation control since all the necessary variables are generated correctly. In such cases, the length of the vectorized JN file is equal to the number of the schema keys. Thus, these coincidences are deemed to comprise a correct output. Otherwise, the tasks are considered failures due to input error during simulation execution. The error rate can be calculated by dividing the number of failed simulations by the total number of simulations. This process entails reading the critical values from the JSON files, vectorizing them, and measuring the lengths of the vectors. By verifying whether the vectors exhibit the expected length, the accurate generation of all necessary variables can be verified, thereby yielding the error rate. All experiments are conducted under identical conditions and the average error rate is calculated over five iterations.

### Experimental results

**Figure 7:**
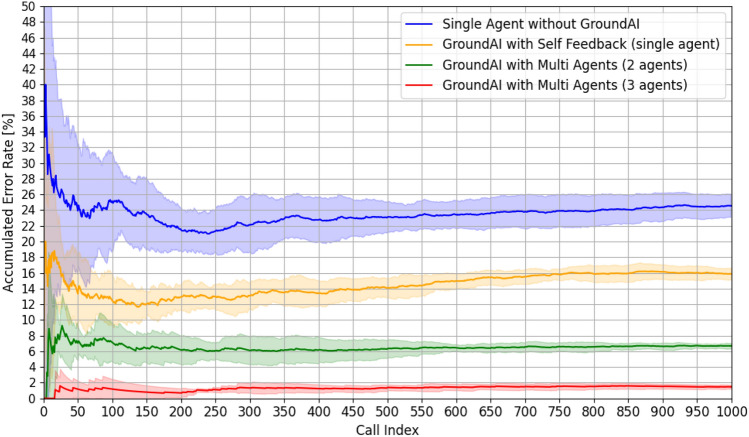
Comparison of accumulated error rates of the four methods over 1000 calls with single variable control.

**Figure 8:**
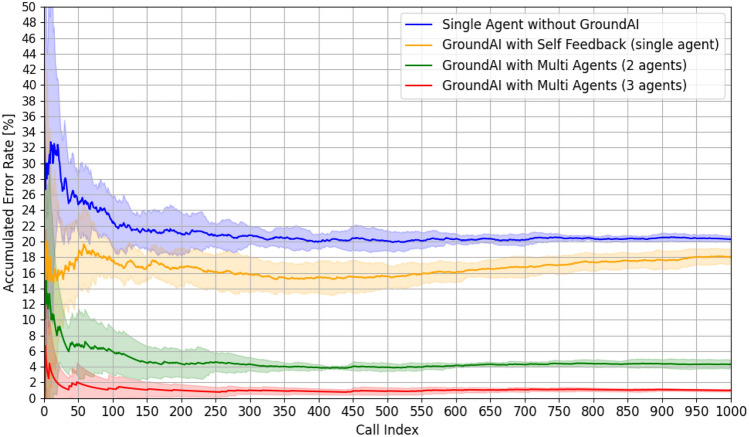
Comparison of accumulated error rates of the four methods over 1000 calls with two-variable control.

In this section, we discuss the methodology and results of measuring error rates by vectorizing the JSON files and checking the lengths of the vectors to determine whether the outputs are generated correctly and can be executed as autonomous ultrasonic simulations via AI orchestration. The overall probability of error occurrence is observed to decrease considerably when Ground AI-based methodologies are employed, particularly in the case of multi-LLM agent approaches, as illustrated in Figs. 7 and 8. The average error rate of the method that does not include Ground AI is 23.89%. In contrast, Ground AI-based methods exhibit markedly lower error rates. In particular, the self-feedback method based on a single-LLM agent exhibits an average error rate of 15.84%, the multi-LLM agent method employing two agents exhibits that of 6.63%, and the multi-LLM agent method utilizing three agents exhibits the lowest average error rate of 1.48%. This suggests that incorporating Ground AI reduces the error rate associated with scenario generation significantly. Further, the multi-agent approaches yield lower error rates than the self-feedback method based on a single-LLM agent, indicating that employing multiple agents enhances the reliability of the simulation control process to a greater extent than the self-review prompt method.

During early phases of repetitive requests (call index: 0–500), the single-LLM agent-based method without Ground AI exhibits a high initial error rate exceeding 26%, which is significantly higher than those of other approaches. Incorporating Ground AI based on the self-feedback method that uses a single-LLM agent to review and correct the answer by itself results in a notable enhancement, with an initial error rate of 16%. However, the multi-LLM agent-based methods based on two or three agents exhibit even more favorable performance, with initial error rates of less than 8% and 4%, respectively.

As the experiments proceed to the accumulated phase (call index: 500–1000), the single-LLM agent-based method without Ground AI exhibits a cumulative error rate exceeding 20%, indicating a lack of sustained performance improvement. In contrast, the Ground AI-based implementation using the self-feedback method exhibits a stabilized error rate of approximately 10%, substantiating the efficacy of Ground AI and self-review mechanisms in a single-agent framework. The performances of the multi-LLM agent-based approaches are even more impressive, with error rates stabilizing below 5% for the two-LLM agent system and below 2% for the three-LLM agent system, which represents the best performance among the compared approaches. As illustrated in Fig. 8, experiments involving the control of two variables demonstrate that the multi-LLM agent approaches maintain impressive performance levels in all cases. Specifically, the error rates stabilize below 5% for the two-LLM agent system and below 2% for the three-LLM agent system, mirroring the patterns observed in the single-variable control experiments.

Further analysis of the standard deviation of error rates highlight the advantages of Ground AI and multi-LLM agent methods. The single-LLM agent without Ground AI exhibits the highest variability between the initial and stabilized stages, reflecting inconsistent performance. In other words, achieving consistent performance with a single-LLM agent without Ground AI is challenging. The Ground AI-based self-feedback method exhibits relatively stable standard deviations—albeit higher than the those of the multi-agent approaches. Notably, the multi-agent approach with three agents exhibits the lowest standard deviations during the early and accumulated phases. This result suggests that the multi-LLM agent-based approaches with Ground AI yield more consistent and reliable performance in long-term practical applications.

The results of this study demonstrate that the integration of Ground AI in self-review and multi-agent systems enhances the efficiency and reliability of solutions for text-based simulation control. The superior performance of the multi-LLM agent-based approach with three agents indicates that employing multiple agents enhances the stability and reliability of autonomous ultrasonic simulation via AI orchestration and improves success rate of adjusting individual parameters during simulation execution. Building on these results, an additional experiment is conducted to explore whether increasing the number of agents beyond three enhances performance further. In the initial experiment, the number of agents is gradually increased up to three, resulting in a reduction of the error rate to 1.48%. Based on this observation, agents are dynamically added until the error is completely eliminated.

### Multi-agent evaluation in a single-variable scenario

**Figure 9:**
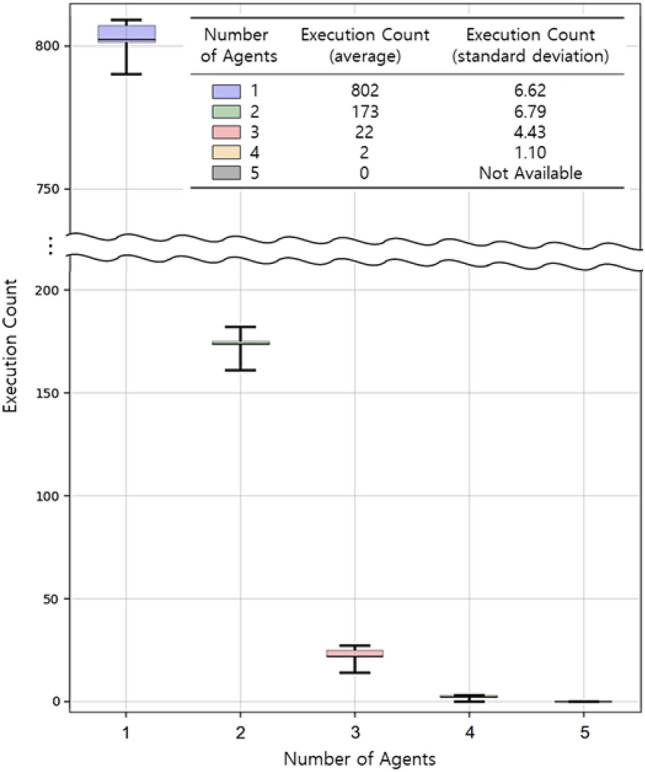
Comparison of empirical results of dynamic agent generation while controlling a single variable.

In this experiment, the error correction capability of a multi-LLM agent system is evaluated by conducting independent trials, where 1000 executions are repeated five times. Each trial is initiated with a single agent, and additional agents are introduced dynamically until the simulation is successfully completed. This approach enables the analysis of the distribution of the number of agents required to generate an error-free output. As illustrated in Fig. 9, the results reveal that, similar to our previous findings, approximately 20% of the scenarios require additional agents. When two agents are employed, 173 scenarios are successfully completed. When three agents are used, 22 scenarios are completed without requiring additional agents. When four or more agents are introduced, all errors are resolved, and no cases required the use of five or more agents. This experiment confirms that a multi-agent LLM system is significantly more robust than a single-agent approach and that, in a single-variable control environment, employing up to four agents is sufficient to eliminate all errors.

### Cost analysis for single-agent vs. multi-agent approaches

Although the primary goal of this study is to reduce errors, resource expenditure also plays a crucial role in selecting an optimal agent configuration. To quantify cost implications in a single-agent environment, 5000 simulation scenarios are considered and all application programming interface (API)-related expenses are recorded. According to the API usage logs provided by OpenAI, the total cost for generating these simulations amount to $37.85 (approximately 38 dollars), translating to an average cost of about $0.00757 (0.76 cents) per simulation. This result serves as a baseline for subsequent cost efficiency analysis of multi-agent systems. Considering that a single agent generates 1000 simulation scenarios in our experiment, the cost for a single-agent architecture is calculated to be approximately $7.57. As the number of agents is increased up to a maximum of five, the costs increase linearly to $7.57, $15.14, $22.71, $30.28, and $37.85, respectively. In other words, although deploying multiple agents reduces scenario generation errors, the cost increases linearly with each additional agent. Therefore, determining the optimal number of agents is crucial to balance cost and efficiency effectively.

## Discussion

The experimental results demonstrate that although the methods integrating Ground AI concepts, particularly the multi-LLM agent approach utilizing three agents, are highly effective in reducing error rates in scenarios where a single variable is controlled, there is potential for further optimization of the performance of the system. Specifically, although the multi-LLM agent approach yields low error rates, further enhancements can be achieved by refining the decision-making mechanisms among the agents. In particular, improvements to the coordination strategies between agents may facilitate more effective consensus and more accurate scenario generation, thereby reducing residual errors and increasing the efficiency of the system. In particular, the multi-LLM agent-based approach with three agents is identified as the most effective method in our experiments in simulation scenarios involving the control of a single variable. However, further reliable autonomous ultrasonic simulation can be realized based on improvements to the decision-making processes among the multi-LLM agent system with many LLM agents and incorporating additional evaluation metrics. This would further enhance the robustness and reliability of Ground AI-based approaches for text-based simulation control in complex systems where efficiency and accuracy are paramount.

Moreover, the experiments in this study employ a vectorization process to measure error rates based on the verification of lengths of the vectors in the JSON files. Although this approach has been demonstrated to be effective, alternative evaluation metrics and methodologies should be considered to assess the ability of the system to produce outputs aligned with the intended objectives. Relying on a single metric may only partially capture the nuances of generative outputs. Thus, future research should explore complementary validation methods, such as expert evaluation, consistency analysis, and functional accuracy assessment. Expert evaluation, particularly based on human-in-the-loop assessment, may provide deeper insights into the contextual validity and reliability of the generated scenarios. Additionally, consistency testing, which involves measuring variations across repeated executions, could offer a quantitative measure of robustness. Further, functional accuracy analysis, comparing LLM-generated commands against predefined ground truth datasets, could enhance the evaluation framework, ensuring that outputs are aligned with expected behaviors.

As the number of agents is increased, the multi-LLM agent-based method exhibits enhanced reliability relative to the self-feedback method based on the single-LLM agent. These results demonstrate that the combination of self-review and multi-LLM agent approaches as Ground AI orchestration has the potential to enhance the scalability and reliability of the simulation control process. These methods address the limitations of relying on the output of a single LLM agent by providing mechanisms for error correction and leveraging multiple independent responses. This not only leverages the diversity of outputs obtained from multiple agents but also automates the validation process, increases efficiency, and reduces the scenario generation error rate. significantly.

## Future works

Our immediate research priority is to develop an integrated validation framework that combines expert evaluation, consistency analysis, and functional accuracy assessment. Rather than merely exploring these methods individually, we will focus on creating a comprehensive system that automatically selects and applies the most appropriate validation technique based on simulation context. This framework will incorporate human-in-the-loop assessment protocols for high-stakes simulations while utilizing automated validation for routine tasks, creating a balanced approach that maximizes both efficiency and reliability across different operational requirements. To enhance scalability and stability for real-time simulations, we plan to investigate the integration of local LLMs, which would reduce dependency on API connections and internet connectivity. This approach would enable more responsive system performance and greater operational autonomy, particularly in environments with limited or unreliable network access, while also potentially reducing latency issues that could impact time-sensitive simulation applications in industrial settings.

We aim to strengthen the Ground AI concept by enhancing the agent paradigm with domain-specific capabilities rather than simply utilizing specialized LLMs. By developing specialized agents with distinct roles and expertise in fields such as nuclear energy, mechanical engineering, and robotics, we can create a more sophisticated multi-agent ecosystem. These domain-specialized agents would possess not only relevant knowledge but also specific reasoning patterns and validation protocols tailored to their respective domains. This approach focuses on the functional specialization of agents within the orchestration architecture, enabling more organized collaboration and domain-appropriate decision-making during simulation tasks. Building upon this agent specialization, we will develop an adaptive orchestration mechanism that dynamically configures the multi-agent system based on both task complexity and domain characteristics. This mechanism will intelligently determine the optimal composition of specialized agents, their interaction patterns, and the most effective validation strategy for each simulation task. By creating this context-aware orchestration layer, we can address the current limitations of fixed validation approaches while maintaining high accuracy standards. The system will learn from past simulation experiences to continuously refine its orchestration decisions, creating a truly adaptive framework that evolves alongside changing simulation requirements and domain knowledge.

## Conclusions

In this study, we propose and implement a text-based simulation control architecture utilizing GPT-4o to enhance the efficiency and effectiveness of ultrasonic simulation control. By modularizing the functionalities of the SimNDT program into discrete functions and enabling simulation control based on natural language commands, the average time required for simulation configuration is reduced significantly—from two minutes to approximately 30 seconds. This indicates a 75% reduction in data generation costs. This improvement illustrates the potential for AI-driven methodologies to facilitate the optimization of simulation processes. To address inherent limitations in LLM-based scenario generation, we introduced the Ground AI method, which integrates self-review mechanisms and multi-agent collaboration for enhanced reliability. The implementation of this approach enables the system to detect inconsistencies in generated scenarios and regenerate outputs under self-review guidance, significantly reducing the scenario generation error rate from 23.89% to 1.48%. This marked improvement underlines the effectiveness of the Ground AI approach in managing complex simulation tasks and highlights the importance of verification mechanisms when deploying LLMs in technical domains requiring high precision and consistency. Looking ahead, the proposed framework shows promise for real-world industrial settings, particularly in digital twin architectures and other time-sensitive applications. Further research could explore GPU-accelerated or distributed processing to enhance real-time scalability, as well as deeper investigations into reliability and security under practical constraints. By pursuing these avenues, our text-based control method and Ground AI framework can evolve into a more versatile solution for comprehensive and autonomous simulation control.

## Electronic supplementary material

Below is the link to the electronic supplementary material.

Supplementary Material 1

Supplementary Material 2

Supplementary Material 3

Supplementary Material 4

Supplementary Material 5

## Data Availability

The datasets generated and analyzed during the current study were produced using generative AI models. These datasets are available from the corresponding authors upon reasonable request.

## References

[1] Chen, C.-H., Lin, C., Jeng, S.-Y., Lin, H.-Y. & Yu, C.-Y. Using ultrasonic sensors and a knowledge-based neural fuzzy controller for mobile robot navigation control. *Multidiscipl. Digital Publish. Inst.* **10**, 466–466. 10.3390/electronics10040466 (2021).

[2] Duan, A., Victorova, M., Zhao, J., Zheng, Y. & Navarro-Alarcon, D. Ultrasound-guided assistive robots for scoliosis assessment with optimization-based control and variable impedance. *Cornell University*10.48550/arxiv.2203.02126 (2022).

[3] Yan, X. et al. Multi-modal interaction control of ultrasound scanning robots with safe human guidance and contact recovery. *Cornell University*10.48550/arxiv.2302.05685 (2023).

[4] Schrage, M., Medany, M. & Ahmed, D. Ultrasound microrobots with reinforcement learning. *Wiley* **8**, 10.1002/admt.202201702 (2023).

[5] Fang, S., Du, Y., Zhang, Y., Meng, F. & Ang, M. H. Research on robotic compliance control for ultrasonic strengthening of aviation blade surface. *Multidiscipl. Digital Publish. Inst.* **14**, 730–730. 10.3390/mi14040730 (2023). 10.3390/mi14040730 PMC10146381 37420963

[6] Beber, L. et al. A passive variable impedance control strategy with viscoelastic parameters estimation of soft tissues for safe ultrasonography. *Cornell University*10.48550/arxiv.2309.14893 (2023).

[7] Brown, T. et al. Language models are few-shot learners. In Larochelle, H., Ranzato, M., Hadsell, R., Balcan, M. & Lin, H. (eds.) *Advances in Neural Information Processing Systems*, vol. 33, 1877–1901 (Curran Associates, Inc., Virtual, 2020).

[8] OpenAI et al. Gpt-4 technical report (2024). arXiv: 2303.08774.

[9] Devlin, J., Chang, M.-W., Lee, K. & Toutanova, K. BERT: Pre-training of deep bidirectional transformers for language understanding. In Burstein, J., Doran, C. & Solorio, T. (eds.) *Proceedings of the 2019 Conference of the North American Chapter of the Association for Computational Linguistics: Human Language Technologies, Volume 1 (Long and Short Papers)*, vol. 1, 4171–4186 (Association for Computational Linguistics, Minneapolis, Minnesota, 2019).

[10] Molero Martínez, M. SimNDT: Ultrasound NDT simulation tool (2019). Github https://github.com/mmolero/SimNDT.

[11] De Vos, L., Nevens, J., Van Eecke, P. & Beuls, K. Construction grammar and procedural semantics for human-interpretable grounded language processing. *Linguistics Vanguard* (2024).

[12] Van der Zee, D.-J. & Van der Vorst, J. A modeling framework for supply chain simulation: Opportunities for improved decision making. *Decis. Sci.* **36**, 65–95. 10.1111/j.1540-5915.2005.00066.x (2005).

[13] Mustafee, N. & Taylor, S. Speeding up simulation applications using wingrid. *Concurr. Comput. Practice Exp.* **21**, 1504–1523. 10.1002/cpe.1401 (2009).

[14] Vaswani, A. Attention is all you need. *Adv. Neural Inf. Process. Syst.* (2017).

[15] Zheng, S. et al. The AI economist: Improving equality and productivity with AI-driven tax policies. *Res. Pap. Econ.* (2020).

[16] Zheng, S. T., Trott, A., Srinivasa, S., Parkes, D. C. & Socher, R. The AI economist: Taxation policy design via two-level deep multiagent reinforcement learning. *Sci. Adv.* **8**, 10.1126/sciadv.abk2607 (2022). 10.1126/sciadv.abk2607 PMC9067926 35507657

[17] Leiter, C. et al. Chatgpt: A meta-analysis after 2.5 months.. *Mach. Learn. Appl.* **16**, 100541 (2024).

[18] Maddigan, P. & Susnjak, T. Chat2vis: Generating data visualizations via natural language using chatgpt, codex and gpt-3 large language models. *IEEE Access* **11**, 45181–45193 (2023).

[19] Kumar, V., Gleyzer, L., Kahana, A., Shukla, K. & Karniadakis, G. E. Mycrunchgpt: A llm assisted framework for scientific machine learning. *J. Mach. Learn. Model. Comput.* **4** (2023).

[20] Hummer, W. et al. Modelops: Cloud-based lifecycle management for reliable and trusted ai. In *2019 IEEE International Conference on Cloud Engineering (IC2E)*, 113–120 (IEEE, 2019).

[21] Feng, X. et al. Large language model-based human-agent collaboration for complex task solving. In Al-Onaizan, Y., Bansal, M. & Chen, Y.-N. (eds.) *Findings of the Association for Computational Linguistics: EMNLP 2024*, 1336–1357, 10.18653/v1/2024.findings-emnlp.72 (Association for Computational Linguistics, Miami, Florida, USA, 2024).

[22] Wang, C. et al. Lami: Large language models for multi-modal human-robot interaction. In *Extended Abstracts of the CHI Conference on Human Factors in Computing Systems*, 1–10 (2024).

[23] Mehandru, N. et al. Large language models as agents in the clinic (2023). arXiv: 2309.10895. 10.1038/s41746-024-01083-y PMC10991271 38570554

[24] Wei, Y. et al. Editable scene simulation for autonomous driving via collaborative llm-agents. In *Proceedings of the IEEE/CVF Conference on Computer Vision and Pattern Recognition*, 15077–15087 (2024).

[25] Pan, X. et al. Very large-scale multi-agent simulation with LLM-powered agents (2025).

[26] Li, R., Patel, T., Wang, Q. & Du, X. Mlr-copilot: Autonomous machine learning research based on large language models agents. arXiv preprint arXiv:2408.14033 (2024).

[27] Zhang, Y. et al. Towards efficient llm grounding for embodied multi-agent collaboration. *Cornell University*10.48550/arXiv.2405. (2024).

[28] Luan, Z. et al. Automatic robotic development through collaborative framework by large language models. In *2023 China Automation Congress (CAC)*, 7736–7741 (IEEE, 2023).

[29] Ramchurn, S. D., Stein, S. & Jennings, N. R. *Trustworthy human-ai partnerships*10.1016/j.isci.2021.102891 (2021). 10.1016/j.isci.2021.102891 PMC8365362 34430804

[30] Liu, H. et al. Enhancing the llm-based robot manipulation through human-robot collaboration. *Inst. Electr. Electron. Eng.* **9**, 6904–6911. 10.1109/lra.2024.3415931 (2024).

[31] Knox, W. B., Stone, P. & Breazeal, C. Teaching agents with human feedback: a demonstration of the tamer framework. In *Proceedings of the Companion Publication of the 2013 International Conference on Intelligent User Interfaces Companion*, 65–66 (2013).

[32] Park, M., Bong, G., Kim, J. & Kim, G. Structural analysis and design using generative AI. *Struct. Eng. Mech.* **91**, 393–401 (2024).

[33] Dorri, A., Kanhere, S. S. & Jurdak, R. Multi-agent systems: a survey. *IEEE Access* **6**, 28573–28593 (2018).

[34] Panait, L. & Luke, S. Cooperative multi-agent learning: The state of the art. *Auton. Agent. Multi-Agent Syst.* **11**, 387–434 (2005).

[35] Meng, Z., Li, J., Gong, Y. & Juang, B.-H. Adversarial teacher-student learning for unsupervised domain adaptation. In *2018 IEEE International Conference on Acoustics, Speech and Signal Processing (ICASSP)*, vol. 437, 5949–5953 (IEEE Press, Calgary, AB, Canada, 2018).

[36] Filippova, K. Controlled hallucinations: Learning to generate faithfully from noisy data. In Cohn, T., He, Y. & Liu, Y. (eds.) *Findings of the Association for Computational Linguistics: EMNLP 2020*, vol. Findings of the Association for Computational Linguistics: EMNLP 2020, 864–870 (Association for Computational Linguistics, Online, 2020).

[37] Vanna.ai. Vanna.ai: Ai-powered sql generation and optimization (2024).

[38] Wang, X. et al. Self-consistency improves chain of thought reasoning in language models. In *The Eleventh International Conference on Learning Representations* (International Conference on Learning Representations, Kigali, Rwanda, 2023).

