# Supplementary material for: Artificial intelligence orchestration for text-based ultrasonic simulation via self-review by multi-large language model agents
Source: Sci Rep. 2025 Apr 11;15:12474. doi: 10.1038/s41598-025-97498-y (PMC11992045; doi:10.1038/s41598-025-97498-y)
Supplement: Supplementary file 1 — Supplementary Material 1 [file 41598_2025_97498_MOESM1_ESM.pdf]

```
        "type": "object",
        "properties": {
                "path_base": {"type": "string", "description": "Base path for the output files.",
"default":"./outputs/example_01_Defect"},
                "defect_depth": {"type": "number", "description": "Depth of the defect in the material."},
        "defect_width": {"type": "number", "description": "Width of the defect in the material."},
        "defect_height": {"type": "number", "description": "Height of the defect in the material."},
        "snapshot": {"type": "boolean", "description": "Whether to take a snapshot or not."},
        "simulation_settings": {
                        "type": "object",
                        "properties": {
                                "width": {"type": "number", "description": "Width of the simulation area."},
                        "height": {"type": "number", "description": "Height of the simulation area."},
                "pixel_mm": {"type": "number", "description": "Conversion rate from pixels to mm."},
        "label": {"type": "number", "description": "Label identifier for the simulation."},
                        "materials": {
                                "type": "array",
                                "items": {
                                        "type": "object",
                                        "properties": {
                                                "label": {"type": "number", "description": "label number of the material
layer."},                               "material": {"type": "string", "description": "Type of material. All output is in
lowercase."} },

                                        "required": ["label", "material"] } },
                        "interval": {"type": "number", "description": "Interval setting for the simulation.", "default" : "10"},
                        "bc_thickness": {
                                "type": "array",
                                "items": {"type": "number"},
                                "description": "Boundary condition thickness settings.", "default" : "[0, 0, 0, 0]"
                        },
                        "signal_ylim": {
                                "type": "array",
                                "items": {"type": "number"},
                                "description": "Y-axis limits for the signal plot.",
                                "default" : "[-0.00075, 0.00075]" },
                        "boundaries": {
                                "type": "array",
                                "items": { "type": "object",
                                "properties": {
                                        "name": {"type": "string", "description": "Name of the boundary."},
                                        "bc": {"type": "string", "description": "Boundary condition.", "default":"AirLayer"},
                                        "size": {"type": "number", "description": "Size of the boundary."}
                                },
                                 "required": ["name", "bc", "size"]
                                }
                         },
                         "transducers": {
                                 "type": "array",
                                  "items": {
                                         "type": "object",
                                         "properties": {
                                                "name": {"type": "string", "description": "Name of the transducer.",
                                                "default" : "excitation"},
                                                "size": {"type": "number", "description": "Size of the transducer.",
                                                "default" : "5.0"},
                                                "center_offset": {"type": "number", "description": "Center offset of the
                                                transducer.", "default" : "0"},
                                                "border_offset": {"type": "number", "description": "Border offset of the
                                                transducer.", "default" : "0"},
                                                "location": {"type": "string", "description": "Location of the transducer.",
                                                "default" : "Top"},
                                                "point_source": {"type": "boolean", "description": "Whether the
                                                transducer is a point source.", "default":"false"}, "enable_window":
                                                {"type": "boolean", "description": "Whether to enable a window
                                                function.", "default":"false"},
                                                "pzt": {"type": "boolean", "description": "Whether the transducer is PZT
                                                (piezoelectric).", "default":"false"}
                                        },
                                         "required": ["name", "size", "center_offset", "border_offset", "location",
                                        "point_source", "enable_window", "pzt"]
                                 }
                         },
                        "objects": {
                                "type": "array",
                                "items": {
                                        "type": "object",
                                        "properties": {
                                                "type": {"type": "string", "description": "Type of object.", "default" :
"Rectangle"},
```
