# Supplementary material for: Artificial intelligence orchestration for text-based ultrasonic simulation via self-review by multi-large language model agents
Source: Sci Rep. 2025 Apr 11;15:12474. doi: 10.1038/s41598-025-97498-y (PMC11992045; doi:10.1038/s41598-025-97498-y)
Supplement: Supplementary file 2 — Supplementary Material 2 [file 41598_2025_97498_MOESM2_ESM.pdf]

```
                        "x0": {"type": "number", "description": "X-coordinate of the object's
                        origin.", "default":"250"},
                        "y0": {"type": "number", "description": "Y-coordinate of the object's
                        origin.","default":"200"},
                        "width": {"type": "number", "description": "Width of the object.",
                        "default":"10"},
                        "height": {"type": "number", "description": "Height of the object.",
                        "default":"5"},
                        "label": {"type": "number", "description": "Label for the object.",
                        "default":"200"}
                    },
                    "required": ["type", "x0", "y0", "width", "height", "label"]
                }
            },
            "signal": {
                "type": "object",
                "properties": {
                    "name": {"type": "string", "description": "Name of the signal function.", "default":
                    "RaisedCosine"},
                    "amplitude": {"type": "number", "description": "Amplitude of the signal.",
                    "default":"1.0"},
                    "frequency": {"type": "number", "description": "Frequency of the signal.",
                    "default":"500000"},
                    "n_cycles": {"type": "number", "description": "Number of cycles for the signal.",
                    "default":"1"}
                },
                "required": ["name", "amplitude", "frequency", "n_cycles"]
            },
            "simulation": {
                "type": "object",
                "properties": {
                    "time_scale": {"type": "number", "description": "Time scale for the simulation.",
                    "default":"1"},
                    "max_freq": {"type": "number", "description": "Maximum frequency for the
                    simulation.", "default":"2000000"},
                    "point_cycle": {"type": "number", "description": "Points per cycle for the
                    simulation.", "default":"10"},
                    "sim_time": {"type": "number", "description": "Total simulation time.",
                    "default":"0.0012"},
                    "order": {"type": "number", "description": "Order of accuracy for the simulation.",
                    "default":"2"},
                    "device": {"type": "string", "description": "Device used for the simulation. ",
                    "default": "GPU"},
                    "dx_user": {"type": "number", "description": "User-defined spatial step size.",
                    "default": "null"},
                    "dt_user": {"type": "number", "description": "User-defined time step size.",
                    "default": "null"}
                },
                "required": ["time_scale", "max_freq", "point_cycle", "sim_time", "order", "device"]
            },
            "inspection": {
                "type": "object",
                "properties": {
                    "location": {"type": "string", "description": "Location of the inspection.",
                    "default":"Top"},
                    "method": {"type": "string", "description": "Inspection method.", "default":
                    "PulseEcho"},
                    "ini": {"type": "number", "description": "Initial point of the inspection range.",
                    "default":"-50"},
                    "end": {"type": "number", "description": "End point of the inspection range.",
                    "default":"50"},
                    "step": {"type": "number", "description": "Step size for the inspection.",
                    "default":"10"},
                    "theta": {
                        "type": "array",
                        "items": {"type": "number"},
                        "description": "Angles for the inspection method.",
                        "default": "[4.71239, 4.71239]"
                    }
                },
                "required": ["location", "method", "ini", "end", "step", "theta"]
            }
        },
        "required": ["width", "height", "pixel_mm", "label", "materials", "interval", "bc_thickness", "signal_ylim",
"boundaries", "transducers", "objects", "signal", "simulation", "inspection"]
    },
    "plot_settings": {
        "type": "object",
```
