# Supplementary material for: Artificial intelligence orchestration for text-based ultrasonic simulation via self-review by multi-large language model agents
Source: Sci Rep. 2025 Apr 11;15:12474. doi: 10.1038/s41598-025-97498-y (PMC11992045; doi:10.1038/s41598-025-97498-y)
Supplement: Supplementary file 3 — Supplementary Material 3 [file 41598_2025_97498_MOESM3_ESM.pdf]

```
                "properties": {
                        "tx_width": {"type": "number", "description": "Width of the transducer plot."},
                        "defect_list": {
                                "type": "array",
                                "items": {
                                        "type": "object",
                                        "properties": {
                                                "type": {"type": "string", "description": "Type of defect.",
                                                "default":"Rectangle"},
                                                "x0": {"type": "number", "description": "X-coordinate of the defect.",
                                                "default":"0"},
                                                "y0": {"type": "number", "description": "Y-coordinate of the defect.",
                                                "default":"200"},
                                                "width": {"type": "number", "description": "Width of the defect.",
                                                "default":"10"},
                                                "height": {"type": "number", "description": "Height of the defect.",
                                                "default":"5"}
                                        },
                                        "required": ["type", "x0", "y0", "width", "height"]
                                }
                        },
                        "colors": {
                                "type": "object",
                                "properties": {
                                        "tx": {
                                                "type": "array",
                                                "items": {"type": "number"},
                                                "description": "Color of the transducer plot.",
                                                "default":"[0.1, 0.5, 0.8]"
                                        },
                                        "defect": {
                                                "type": "array",
                                                "items": {"type": "number"},
                                                "description": "Color of the defect plot.",
                                                "default":"[0.3, 0.3, 0.3]"
                                        }
                                },
                                "required": ["tx", "defect"]
                        }
                        },
                        "required": ["tx_width", "defect_list", "colors"]
                }
        },
        "required": ["path_base", "defect_depth", "defect_width", "defect_height", "snapshot", "simulation_settings",
        "plot_settings"]
}
```
