# Supplementary material for: Artificial intelligence orchestration for text-based ultrasonic simulation via self-review by multi-large language model agents
Source: Sci Rep. 2025 Apr 11;15:12474. doi: 10.1038/s41598-025-97498-y (PMC11992045; doi:10.1038/s41598-025-97498-y)
Supplement: Supplementary file 5 — Supplementary Material 5 [file 41598_2025_97498_MOESM5_ESM.pdf]

Supplementary Figures

```
        "type": "object",
        "properties": {
                "path_base": {"type": "string", "description": "Base path for the output files.",
"default":"./outputs/example_01_Defect"},
                "defect_depth": {"type": "number", "description": "Depth of the defect in the material."},
        "defect_width": {"type": "number", "description": "Width of the defect in the material."},
        "defect_height": {"type": "number", "description": "Height of the defect in the material."},
        "snapshot": {"type": "boolean", "description": "Whether to take a snapshot or not."},
        "simulation_settings": {
                        "type": "object",
                        "properties": {
                                "width": {"type": "number", "description": "Width of the simulation area."},
                        "height": {"type": "number", "description": "Height of the simulation area."},
                "pixel_mm": {"type": "number", "description": "Conversion rate from pixels to mm."},
         "label": {"type": "number", "description": "Label identifier for the simulation."},
                         "materials": {
                                "type": "array",
                                "items": {
                                        "type": "object",
                                        "properties": {
                                                "label": {"type": "number", "description": "label number of the material
layer."},                               "material": {"type": "string", "description": "Type of material. All output is in
lowercase."} },

                                        "required": ["label", "material"] } },
                        "interval": {"type": "number", "description": "Interval setting for the simulation.", "default" : "10"},
                        "bc_thickness": {
                                "type": "array",
                                "items": {"type": "number"},
                                "description": "Boundary condition thickness settings.", "default" : "[0, 0, 0, 0]"
                        },
                        "signal_ylim": {
                                "type": "array",
                                "items": {"type": "number"},
                                "description": "Y-axis limits for the signal plot.",
                                "default" : "[-0.00075, 0.00075]" },
                        "boundaries": {
                                "type": "array",
                                "items": { "type": "object",
                                "properties": {
                                        "name": {"type": "string", "description": "Name of the boundary."},
                                        "bc": {"type": "string", "description": "Boundary condition.", "default":"AirLayer"},
                                        "size": {"type": "number", "description": "Size of the boundary."}
                                },
                                 "required": ["name", "bc", "size"]
                                }
                         },
                         "transducers": {
                                 "type": "array",
                                  "items": {
                                          "type": "object",
                                          "properties": {
                                                "name": {"type": "string", "description": "Name of the transducer.",
                                                "default" : "excitation"},
                                                "size": {"type": "number", "description": "Size of the transducer.",
                                                "default" : "5.0"},
                                                "center_offset": {"type": "number", "description": "Center offset of the
                                                transducer.", "default" : "0"},
                                                "border_offset": {"type": "number", "description": "Border offset of the
                                                transducer.", "default" : "0"},
                                                "location": {"type": "string", "description": "Location of the transducer.",
                                                "default" : "Top"},
                                                "point_source": {"type": "boolean", "description": "Whether the
                                                transducer is a point source.", "default":"false"}, "enable_window":
                                                {"type": "boolean", "description": "Whether to enable a window
                                                function.", "default":"false"},
                                                "pzt": {"type": "boolean", "description": "Whether the transducer is PZT
                                                (piezoelectric).", "default":"false"}
                                        },
                                         "required": ["name", "size", "center_offset", "border_offset", "location",
                                        "point_source", "enable_window", "pzt"]
                                 }
                         },
                        "objects": {
                                "type": "array",
                                "items": {
                                        "type": "object",
                                        "properties": {
                                                "type": {"type": "string", "description": "Type of object.", "default" :
"Rectangle"},
```

Figure S1. Schema structure for simulation configurations used in GPT-4o (part 1 of 3).

```
                    "x0": {"type": "number", "description": "X-coordinate of the object's
                    origin.", "default":"250"},
                    "y0": {"type": "number", "description": "Y-coordinate of the object's
                    origin.","default":"200"},
                    "width": {"type": "number", "description": "Width of the object.",
                    "default":"10"},
                    "height": {"type": "number", "description": "Height of the object.",
                    "default":"5"},
                    "label": {"type": "number", "description": "Label for the object.",
                    "default":"200"}
                },
                "required": ["type", "x0", "y0", "width", "height", "label"]
            }
        },
        "signal": {
            "type": "object",
            "properties": {
                "name": {"type": "string", "description": "Name of the signal function.", "default":
                "RaisedCosine"},
                "amplitude": {"type": "number", "description": "Amplitude of the signal.",
                "default":"1.0"},
                "frequency": {"type": "number", "description": "Frequency of the signal.",
                "default":"500000"},
                "n_cycles": {"type": "number", "description": "Number of cycles for the signal.",
                "default":"1"}
            },
            "required": ["name", "amplitude", "frequency", "n_cycles"]
        },
        "simulation": {
            "type": "object",
            "properties": {
                "time_scale": {"type": "number", "description": "Time scale for the simulation.",
                "default":"1"},
                "max_freq": {"type": "number", "description": "Maximum frequency for the
                simulation.", "default":"2000000"},
                "point_cycle": {"type": "number", "description": "Points per cycle for the
                simulation.", "default":"10"},
                "sim_time": {"type": "number", "description": "Total simulation time.",
                "default":"0.0012"},
                "order": {"type": "number", "description": "Order of accuracy for the simulation.",
                "default":"2"},
                "device": {"type": "string", "description": "Device used for the simulation. ",
                "default": "GPU"},
                "dx_user": {"type": "number", "description": "User-defined spatial step size.",
                "default": "null"},
                "dt_user": {"type": "number", "description": "User-defined time step size.",
                "default": "null"}
            },
            "required": ["time_scale", "max_freq", "point_cycle", "sim_time", "order", "device"]
        },
        "inspection": {
            "type": "object",
            "properties": {
                "location": {"type": "string", "description": "Location of the inspection.",
                "default":"Top"},
                "method": {"type": "string", "description": "Inspection method.", "default":
                "PulseEcho"},
                "ini": {"type": "number", "description": "Initial point of the inspection range.",
                "default":"-50"},
                "end": {"type": "number", "description": "End point of the inspection range.",
                "default":"50"},
                "step": {"type": "number", "description": "Step size for the inspection.",
                "default":"10"},
                "theta": {
                    "type": "array",
                    "items": {"type": "number"},
                    "description": "Angles for the inspection method.",
                    "default": "[4.71239, 4.71239]"
                }
            },
            "required": ["location", "method", "ini", "end", "step", "theta"]
        }
    },
    "required": ["width", "height", "pixel_mm", "label", "materials", "interval", "bc_thickness", "signal_ylim",
"boundaries", "transducers", "objects", "signal", "simulation", "inspection"]
},
"plot_settings": {
    "type": "object",
```


Figure S2. Schema structure for simulation configurations used in GPT-4o (part 2 of 3).

```
                "properties": {
                    "tx_width": {"type": "number", "description": "Width of the transducer plot."},
                    "defect_list": {
                        "type": "array",
                        "items": {
                            "type": "object",
                            "properties": {
                                "type": {"type": "string", "description": "Type of defect.",
                                "default":"Rectangle"},
                                "x0": {"type": "number", "description": "X-coordinate of the defect.",
                                "default":"0"},
                                "y0": {"type": "number", "description": "Y-coordinate of the defect.",
                                "default":"200"},
                                "width": {"type": "number", "description": "Width of the defect.",
                                "default":"10"},
                                "height": {"type": "number", "description": "Height of the defect.",
                                "default":"5"}
                            },
                            "required": ["type", "x0", "y0", "width", "height"]
                        }
                    },
                    "colors": {
                        "type": "object",
                        "properties": {
                            "tx": {
                                "type": "array",
                                "items": {"type": "number"},
                                "description": "Color of the transducer plot.",
                                "default":"[0.1, 0.5, 0.8]"
                            },
                            "defect": {
                                "type": "array",
                                "items": {"type": "number"},
                                "description": "Color of the defect plot.",
                                "default":"[0.3, 0.3, 0.3]"
                            }
                        },
                        "required": ["tx", "defect"]
                    }
                },
                "required": ["tx_width", "defect_list", "colors"]
            }
        },
        "required": ["path_base", "defect_depth", "defect_width", "defect_height", "snapshot", "simulation_settings",
        "plot_settings"]
}
```


Figure S3. Schema structure for simulation configurations used in GPT-4o (part 3 of 3).
